# *Plasmodium knowlesi* Malaria in Persons Returning to Israel from Thailand, 2023

**DOI:** 10.3201/eid3107.250444

**Published:** 2025-07

**Authors:** Yael Paran, Ami Neuberger, Muna Massarwa, Maisam Amar, Julia Vainer, Moran Szwarcwort Cohen, Ohad Shalom, Shirly Elbaz, Maya Davidovich, Oscar David Kirstein, Tamar Grossman

**Affiliations:** Tel Aviv Sourasky Medical Center, Tel Aviv, Israel (Y. Paran); Division of Internal Medicine, Rambam Health Care Campus, Haifa, Israel (A. Neuberger, M. Massarwa); Technion Faculty of Medicine, Haifa (A. Neuberger, M. Amar); Infectious Disease Unit, Lady Davis Carmel Medical Center, Haifa (M. Amar); Public Health Laboratories–Jerusalem, Public Health Services, Ministry of Health, Jerusalem, Israel (J. Vainer, S. Elbaz, M. Davidovich, O.D. Kirstein, T. Grossman); Microbiology Laboratory, Rambam Health Care Campus, Haifa (M.S. Cohen); Microbiology Laboratory, Tel Aviv Sourasky Medical Center, Tel Aviv (O. Shalom)

**Keywords:** Plasmodium knowlesi, malaria, parasites, travelers, zoonoses, Pkmsp1, Thailand

## Abstract

We describe 2 cases of *Plasmodium knowlesi* malaria in persons from Israel who traveled to Thailand in 2023. One patient, likely infected in northwestern Thailand, might signal geographic expansion into areas not previously associated with human infection. The infection’s rarity in travelers, diagnostic challenges, and potential severity underscore the importance of clinical awareness.

*Plasmodium knowlesi*, known as the fifth human malaria parasite, is a zoonotic malaria species maintained in a sylvatic cycle involving long-tailed (*Macaca fascicularis*) and pig-tailed (*Macaca nemestrina*) macaques and *Anopheles* (*Cellia*) *leucosphyrus* mosquitoes (*1*). In Thailand, anthroponotic malaria cases have decreased because of intervention programs, whereas *P. knowlesi* infections have increased, raising public health concerns (*2*,*3*). We report 2 cases of *P. knowlesi* malaria in persons from Israel who traveled to Thailand in 2023. Patient 1 likely acquired malaria in northwestern Thailand and might represent a sentinel case for geographic expansion. Patient 2 was infected in a recognized endemic focus.

In July 2023, a 25-year-old man (patient 1) sought care for fever, chills, nausea, and retro-orbital pain 1 day after returning from a 7-month trip across Southeast Asia. He spent his final month in northern Thailand, beginning in Chiang Mai and ending with 12 days in Pai, Mae Hong Son Province (Figure 1). He reported jungle trekking, monkey sightings, and no use of malaria prophylaxis. An initial rapid diagnostic test for detecting histidine-rich protein 2 and aldolase was negative, and another for detecting histidine-rich protein 2 and lactate dehydrogenase was weakly positive. A thick smear was initially negative, but a repeat smear 3 hours later revealed nonspeciated *Plasmodium*. Laboratory findings included leukopenia, thrombocytopenia, increased bilirubin and transaminase levels, and increased C-reactive protein level. TaqMan real-time PCR targeting the 18S rDNA gene performed at the Parasitology Reference Laboratory (https://www.gov.il/he/departments/units/parasite-reference-lab) confirmed *P. knowlesi* (*4*,*5*). PCR and 18S rDNA sequencing yielded a 939-bp fragment with 99.36% identity to *P. knowlesi* strain H in PlasmoDB (https://plasmodb.org/plasmo/app/record/gene/PKNH_0320900). We treated the patient with artemether/lumefantrine (80 mg/480 mg at 0 and 8 hours and then every 12 hours for a total of 6 doses), and he fully recovered without recrudescence.

**Figure 1:**
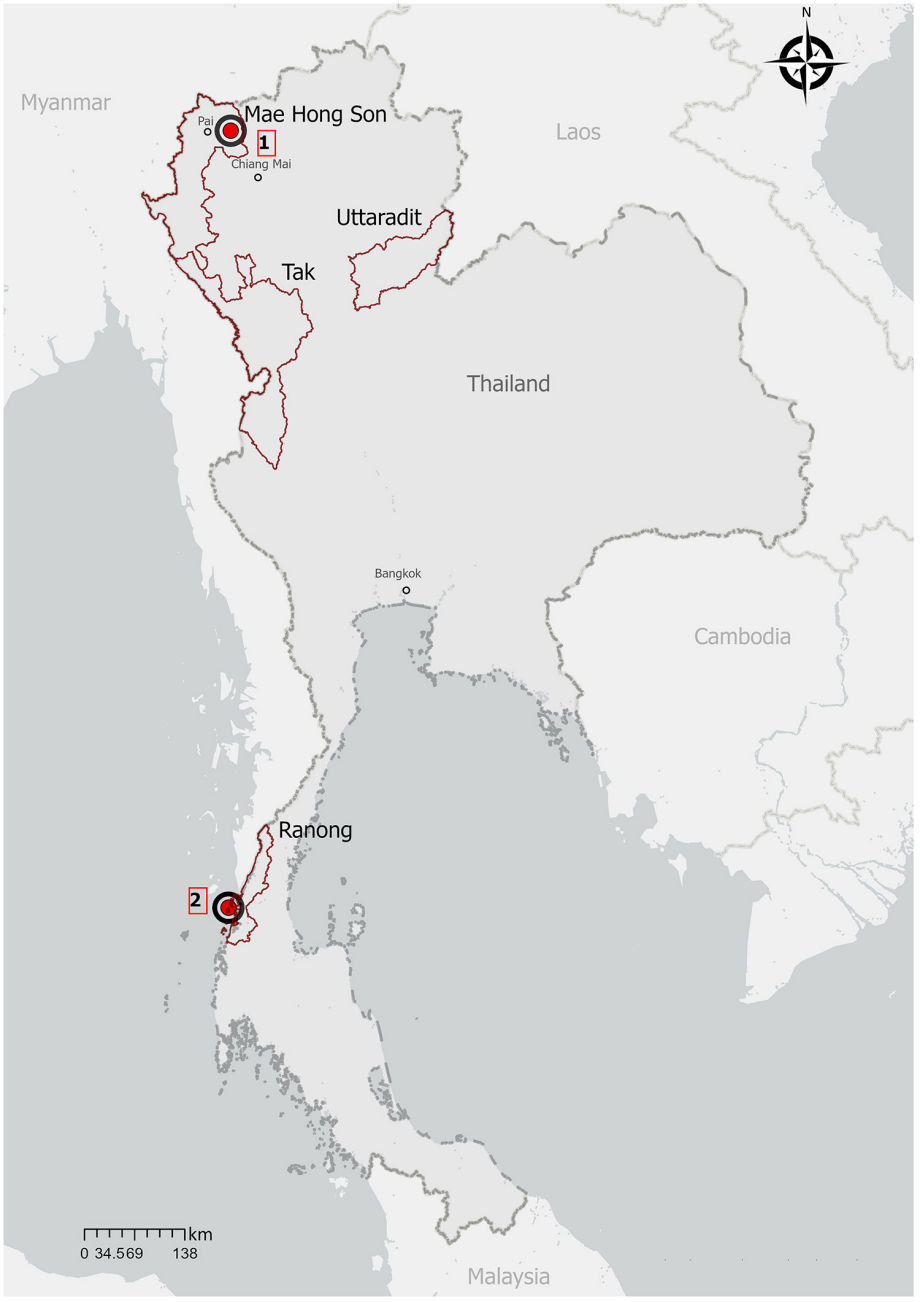
Travel locations of persons with *Plasmodium knowlesi* malaria returning to Israel from Thailand, 2023. Patient 1 might represent a sentinel case of human *P. knowlesi* infection acquired in northern Thailand. Patient 2 visited Koh Phayam Island in Ranong Province in southern Thailand, a recognized endemic area near the Malaysia border. Map created with ArcGIS Pro software version 3.3.2 (ESRI, https://www.esri.com).

In March 2023, we evaluated a 34-year-old man (patient 2), who had spent 6 months in Southeast Asia, for *P. knowlesi* by using the same molecular diagnostics. He spent his last 2 weeks on Koh Phayam Island in Ranong Province, a recognized endemic area (Figure 1) (*2*). He reported jungle hiking and macaque contact and did not use prophylaxis. We also treated patient 2 with standard oral artemether/lumefantrine, and he fully recovered.

We sequenced the polymorphic C-terminal region of the *Pkmsp1* gene to explore genetic diversity. The sequence from patient 1 (northern Thailand) showed 1 synonymous mutation, differentiating it from the *P. knowlesi* H strain in PlasmoDB (https://plasmodb.org/plasmo/app/record/gene/PKNH_0728900). The closest sequences were from Malaysia, consistent with shared ancestry (*6*). The translated amino acid sequence matched haplotype H3, which was similar to an amino acid sequence predicted from isolate JF837348 from southern Thailand. The isolate from patient 2 (southern Thailand) was identical to isolate JF837351 from southern Thailand and matched haplotype H2 that is common in the region (*6**,**7*) (Figure 2). 

**Figure 2:**
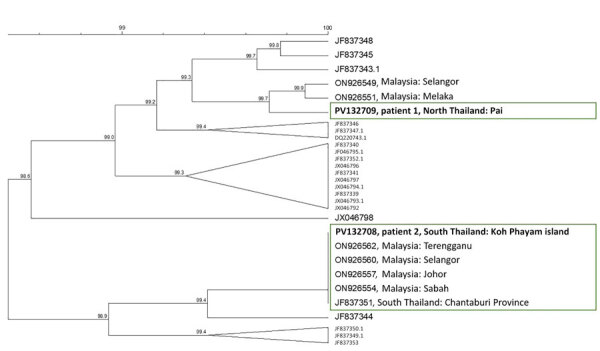
Phylogenetic analysis of *Pkmsp1* sequences for *Plasmodium knowlesi* malaria in persons returning to Israel from Thailand, 2023. Dendogram was based on an 850-bp fragment of the C-terminal region of *Pkmsp1*, including all published sequences from Thailand, the isolates from patients 1 and 2 in this study (bold text), and the most closely related sequences identified through BLAST (https://blast.ncbi.nlm.nih.gov) analysis. No exact matches were identified for patient 1 (northern Thailand); the geographic origin of 2 closely related sequences is indicated. For patient 2 (southern Thailand), several identical sequences were found in GenBank (green box). Accession numbers are given. Clustering analysis was performed by using the unweighted pair group with arithmetic mean method with open gap penalty set to 100% and unit gap penalty set to 0%, without correction. Scale bar indicates percentage similarity between sequences.

The Institutional Review Board (Helsinki Committee, https://www.tasmc.org.il/rd/en/next-unit/helsinki-committee) of Tel Aviv Sourasky Medical Center waived ethical approval. We deposited sequences in GenBank (patient 1: 18S rDNA, accession no. PV123279, and *Pkmsp1*, accession no. PV132709; patient 2: 18S rDNA, accession no. PV123278, and *Pkmsp1*, accession no. PV132708). 

The likely exposure of patient 1 in Pai or Chiang Mai represents the northernmost documented location of *P. knowlesi* human infection in Thailand. Previously, the northernmost reported endemic cases were in Tak Province, bordering Myanmar and Uttaradit province, near the Laos border (*8*). The genetic divergence of the isolate from patient 1 might reflect local parasite diversity or the limited sequence data from northern Thailand and neighboring regions. The Pkmsp1 protein is subject to purifying selection by the host immune system, with surviving variants potentially representing fitter strains. Ongoing genomic surveillance is needed to determine whether patient 1 reflects true local transmission or indicates a broader haplotype range in the region.

Although >280,000 Israelis visited Thailand in 2023, no other confirmed malaria cases were reported among returning travelers, and no other *P. knowlesi* cases have been reported in Israel in >20 years. In addition to its rarity in travelers, *P. knowlesi* malaria poses diagnostic challenges because of rapid diagnostic test insensitivity and morphologic similarity to other *Plasmodium* species, increasing the likelihood of underdiagnosis. Parasitemia levels might be low early in infection, but clinicians should remain alert to the potential for rapid progression to hyperparasitemia, which is a key risk factor for severe malaria attributed to the ability of *P. knowlesi* to infect all erythrocyte stages and its short asexual replication cycle (*9*,*10*). In Malaysia, severe malaria occurred in 6%–9% of cases in district hospitals and up to 29% in tertiary care (*9*).

Both patients reported here met Centers for Disease Control and Prevention criteria for chemoprophylaxis, having stayed in forested regions near international borders (https://wwwnc.cdc.gov/travel/yellowbook/2024/preparing/yellow-fever-vaccine-malaria-prevention-by-country#6419). The patients’ extended travel and jungle exposure highlight the importance of itinerary-specific pretravel counseling.

In conclusion, *P. knowlesi* malaria in patient 1 might represent early evidence of zoonotic spillover of *P. knowlesi* in a new area (northern Thailand). Traveler-based surveillance and molecular tools can provide early warning of geographic shifts in malaria transmission. Heightened clinical awareness and improved diagnostics are essential for timely detection and control.

## References

[1] Singh B, Kim Sung L, Matusop A, Radhakrishnan A, Shamsul SS, Cox-Singh J, et al. A large focus of naturally acquired *Plasmodium knowlesi* infections in human beings. Lancet. 2004;363:1017–24. 10.1016/S0140-6736(04)15836-4 15051281

[2] US President’s Malaria Initiative. Thailand malaria profile: FY 2024. Washington (DC): US Agency for International Development; 2024.

[3] World Health Organization. World Malaria Report 2024: addressing inequity in the global malaria response. Geneva: The Organization; 2024.

[4] Divis PC, Shokoples SE, Singh B, Yanow SK. A TaqMan real-time PCR assay for the detection and quantitation of *Plasmodium knowlesi.* Malar J. 2010;9:344. 10.1186/1475-2875-9-344 21114872 PMC3009662

[5] Shokoples SE, Ndao M, Kowalewska-Grochowska K, Yanow SK. Multiplexed real-time PCR assay for discrimination of *Plasmodium* species with improved sensitivity for mixed infections. J Clin Microbiol. 2009;47:975–80. 10.1128/JCM.01858-08 19244467 PMC2668309

[6] Tapaopong P, Chainarin S, Mala A, Rannarong A, Kangkasikorn N, Kusolsuk T, et al. Declining genetic polymorphism of the C-terminus Merozoite Surface Protein-1 amidst increased *Plasmodium knowlesi* transmission in Thailand. Malar J. 2024;23:342. 10.1186/s12936-024-05162-z 39538241 PMC11562464

[7] Jongwutiwes S, Buppan P, Kosuvin R, Seethamchai S, Pattanawong U, Sirichaisinthop J, et al. *Plasmodium knowlesi* Malaria in humans and macaques, Thailand. Emerg Infect Dis. 2011;17:1799–806. 10.3201/eid1710.110349 22000348 PMC3310673

[8] Narapakdeesakul D, Pengsakul T, Kaewparuehaschai M, Thongsahuan S, Moonmake S, Lekcharoen P, et al. Zoonotic simian malaria parasites in free-ranging *Macaca fascicularis* macaques and human malaria patients in Thailand, with a note on genetic characterization of recent isolates. Acta Trop. 2023;248:107030. 10.1016/j.actatropica.2023.107030 37742788

[9] Barber BE, Grigg MJ, Cooper DJ, van Schalkwyk DA, William T, Rajahram GS, et al. Clinical management of *Plasmodium knowlesi* malaria. Adv Parasitol. 2021;113:45–76. 10.1016/bs.apar.2021.08.004 34620385 PMC9299581

[10] Lee WC, Cheong FW, Amir A, Lai MY, Tan JH, Phang WK, et al. *Plasmodium knowlesi*: the game changer for malaria eradication. Malar J. 2022;21:140. 10.1186/s12936-022-04131-8 35505339 PMC9066973

